# Influence of Zirconium on the Microstructure, Selected Mechanical Properties, and Corrosion Resistance of Ti_20_Ta_20_Nb_20_(HfMo)_20−x_Zr_x_ High-Entropy Alloys

**DOI:** 10.3390/ma17112730

**Published:** 2024-06-04

**Authors:** Karsten Glowka, Maciej Zubko, Paweł Świec, Krystian Prusik, Magdalena Szklarska, Danuta Stróż

**Affiliations:** 1 Institute of Materials Engineering, University of Silesia in Katowice, 75 Pułku Piechoty 1A St., 41-500 Chorzów, Poland; karsten.glowka@us.edu.pl (K.G.); pawel.swiec@us.edu.pl (P.Ś.); krystian.prusik@us.edu.pl (K.P.); magdalena.szklarska@us.edu.pl (M.S.); danuta.stroz@us.edu.pl (D.S.); 2 Faculty of Science, Department of Physics, University of Hradec Králové, Rokitanského 62, 500 03 Hradec Králové, Czech Republic

**Keywords:** high-entropy alloys, multi-component alloys, microstructure, mechanical properties, corrosion resistance

## Abstract

The presented work considers the influence of the hafnium and molybdenum to zirconium ratio of Ti_20_Ta_20_Nb_20_(HfMo)_20−x_Zr_x_ (where x = 0, 5, 10, 15, 20 at.%) high-entropy alloys in an as-cast state for potential biomedical applications. The current research continues with our previous results of hafnium’s and molybdenum’s influence on a similar chemical composition. In the presented study, the microstructure, selected mechanical properties, and corrosion resistance were investigated. The phase formation thermodynamical calculations were also applied to predict solid solution formation after solidification. The calculations predicted the presence of multi-phase, body-centred cubic phases, confirmed using X-ray diffraction and scanning electron microscopy. The chemical composition analysis showed the segregation of alloying elements. Microhardness measurements revealed a decrease in microhardness with increased zirconium content in the studied alloys. The corrosion resistance was determined in Ringer’s solution to be higher than that of commercially applied biomaterials. The comparison of the obtained results with previously reported data is also presented and discussed in the presented study.

## 1. Introduction

The human body is a complex system characterised by countless interconnected physical, chemical, biological, and mechanical phenomena. These processes are essential for maintaining homeostasis and overall health. Scientists face significant challenges in understanding how to maintain these complex systems, particularly when disruptions occur due to diseases or ageing. Particularly, the degeneration of components within the musculoskeletal system can be intensified by factors such as age and body weight. In response to such challenges, the field of materials engineering has provided significant advancements, especially in the development of biomedical implants. Metallic implants represent a critical category of materials extensively used for medical applications [1]. According to the literature data, the selection and performance of these implants are based on several key properties, including chemical composition, mechanical strength, wear resistance, corrosion resistance, and the possibility of surface characteristic modifications, such as roughness or porosity [2]. These attributes are essential for ensuring the endurance and compatibility of implants in both short-term and long-term medical applications. The literature sources provide that gold plates used for covering an open skull were also applied by ancient civilisations for advanced surgeries like cranioplasty [3,4]. Historical sources also describe the application of golden bridges or gold/copper dental implants in ancient Egypt [5,6]. In 1562, golden plates were also applied in the cleft palate treatment process [7]. Further investigations of other elements in order to determine their medical applications were the fundamentals of biocompatibility phenomena. Based on the obtained results, it was concluded that the group of biocompatibility elements was limited to Ti, Zr, Nb, Ta, Au, and Sn [8]. According to the literature data, Ti and Ti-based alloys exhibit the highest application ability in medicine for short- and long-term applications [1]. The structure of titanium alloys at different temperatures showcases two predominant phases: the low-temperature hexagonal close-packed (α-phase, *HCP*) and the high-temperature body-centred cubic (β-phase, *BCC*) [9]. Several alloying elements could stabilise each of the phases [8]. However, the most widely investigated group of Ti-based medical alloys are β-Ti alloys due to the improvement of mechanical properties and corrosion resistance. Additionally, for β phase Ti-based alloys, the lower Young’s modulus could be a crucial advantage, especially for bone implant applications.

In the case of the α phase, commercially pure titanium (cp-Ti) Grades 1–4 are widely used metallic biomaterials in medical applications [10]. Cp-Ti Grade 4 possesses the highest tensile strength (550 MPa) and yield strength (485 MPa). On the other hand, the highest elongation was measured for the first generation (Grade 1), which was equal to 24%. Very important, especially for implant application, is that the elastic modulus was the lowest and is equal to 102.7 GPa for cp-Ti Grade 1 and Grade 2 compared to human bone (30 GPa) [10,11]. For cp-Ti alloys, the presence of a self-passive titanium dioxide (TiO_2_) layer ensured high corrosion resistance in different simulated body fluid environments [12,13]. Due to the presented properties of commercially pure titanium, their applications include dental implants [14]. 

A widely medical-used example of a Ti-based alloy with a mixture of α + β phases is Ti-6Al-4V (Ti64) [10]. The presence of a mixture of α + β phases is associated with the Al (α-stabiliser) and V (β-stabiliser) alloying elements. Considering the biocompatibility of Ti-6Al-4V alloys, the selection of aluminium and vanadium can be discussed. Biocompatibility measurements of both elements revealed that V in the elemental state is entirely toxic for the human body, and Al contributes to the progress of Alzheimer’s disease and epilepsy [15]. Despite these concerns, Ti64 alloys are extensively used in dental implants and hip replacements [16,17] due to the improvement of mechanical properties and corrosion resistance in comparison to cp-Ti [10,18]. Further explorations in Ti-based alloys have led to the development of other α + β phases multi-component systems, such as Ti-6Al-7Nb, Ti-5Al-2.5Fe and Ti-15Sn-4Nb-2Ta-0.2Pd, among others. However, the low biocompatibility of Al, B, Fe, and Pd should be taken into account, and further processing of the mentioned alloys, such as annealing or ageing, is necessary to obtain more favourable materials from a biomedical point of view in the *BCC* β phase [8,10].

Some examples of biomedical β-Ti alloys include binary Ti-15Mo, ternary Ti-16Nb-9.5Hf (Tiadyne 1610), Ti-13Nb-13Zr, and multi-component Si-containing Ti-15Mo-2.8Nb-0.2Si (21SRx), Fe-containing Ti-12Mo-6Zr-2Fe (TMZF), Al-containing Ti-15Mo-5Zr-3Al, Ti-35.3Nb-5.1Ta-7.1Zr, and Ti-29Nb-13Ta-4.6Zr alloys [10,19]. In the investigation of various Ti-based alloys for medical applications, mechanical properties, such as tensile strength, yield strength, and elongation, play crucial roles in determining suitability and performance. Ti-35.3Nb-5.1Ta-7.1Zr alloys demonstrate the lowest tensile strength among the discussed materials, at 596.7 MPa. On the other hand, the annealed TMZF (Ti-12Mo-6Zr-2Fe) and aged Ti-15Mo-5Zr-3Al alloys exhibit the highest tensile strength, both reaching 1100 MPa. The lowest value of 544 MPa was recorded for the annealed binary Ti-15Mo alloy for yield strength, indicating its lower resistance to deformation under load. On the other hand, the highest yield strength, at 1060 MPa, was found in similar alloys that underwent thermal treatment, showing enhanced structural integrity after processing. The aged Ti-13Nb-13Zr and Hf-containing Tiadyne 1610 alloys recorded the lowest value at 10%. This suggests a reduced ability to undergo deformation without fracturing, which may limit their use in applications requiring high flexibility. Remarkably, the Ti-15Mo-5Zr-3Al alloy, containing aluminium, showed an elongation of 25%, reflecting better ductility, possibly due to aluminium’s influence on the microstructure. A Young’s modulus closest to the human bone (30 GPa) was observed for the Ti-35.3Nb-5.1Ta-7.1Zr alloy (55.5 GPa) [10,11]. Additionally, high corrosion resistance was also observed. For example, for ternary Ti-13Nb-13Zr, the corrosion potential (E_corr_) measured in Ringer’s solution was −609 mV vs. SCE (where SCE is saturated calomel electrode) [20]. Examples of medical applications of β-Ti alloys include dental implants, bones, joints, teeth prosthesis, surgical bone cement, and advanced cemented and cementless artificial hip joints [21,22,23].

High-entropy alloys (HEAs), classified under multi-principal elemental alloys (MPEAs), have emerged as a significant area of interest in materials science, particularly for their potential in biomedical applications.

Biomedical high-entropy alloys (bio-HEAs) are mainly composed of five or six elements, such as Ti, Ta, Nb, Zr, Mo, and Hf, which are also denoted as β-stabilisers in Ti-based alloys [24,25,26,27,28]. The literature data indicate that bio-HEAs are mainly produced by arc melting or mechanical alloying techniques [29]. According to the literature data, the biocompatibility of Mo and Hf is still being discussed [8]. However, Mo is widely used as an alloying element in Ti-based alloys, and the presence of a stable MoO_3_ oxide layer ensures the high corrosion resistance of pure Mo [30,31]. 

Recent studies on bio-HEAs containing five or six elements, such as Ti-Ta-Nb-Zr-Mo, Ti-Ta-Nb-Zr-Hf, and Ti-Ta-Nb-Zr-Mo-Hf, have shown promising results. These alloys have been confirmed to predominantly exhibit single- or dual-*BCC* phase structures [26,27,28,32]. An analysis of their microstructures highlighted the presence of dendritic and interdendritic regions, with notable segregations of alloying elements in each area, indicating complex internal structures that potentially influence mechanical properties [27,28,33,34]. For Mo- and/or Hf-containing bio-HEAs, mechanical property measurements underlined high compressive yield strength and plasticity [26,32]. Furthermore, these alloys exhibit superior corrosion and biocorrosion resistance in simulated body fluid environments. This resistance is largely attributed to the formation of stable oxide layers on the alloying elements, which protect the underlying metal from degradation. This characteristic is particularly important in medical implants, where material longevity and stability in hostile biological environments are crucial [1,26,27,28,32,35]. Regarding biocompatibility, these bio-HEAs have also been found to outperform conventional biomedical materials, such as Ti-6Al-4V. The higher level of biocompatibility observed suggests that these materials are less likely to cause adverse biological reactions and are more compatible with human tissue. This makes them highly suitable for a variety of implantable medical devices, offering a potential advancement over traditional titanium-based alloys [36]. 

In the present work, novel Ti_20_Ta_20_Nb_20_(HfMo)_20−x_Zr_x_ (where x = 0, 5, 10, 15, and 20 at.%) alloys have been designed and investigated in an as-cast state as potential biomedical high-entropy materials. In the presented study, the main aim was to determine the influence of the Zr/(HfMo) ratio on the structure, mechanical properties, and corrosion resistance of the obtained alloys. Additionally, in our previous works, the influence of Mo/(ZrHf) and Hf/(ZrMo) on the phase formation, microstructure, selected mechanical properties, and corrosion resistance were investigated [27,28]. For Mo- and Hf-containing bio-HEAs, the XRD revealed the presence of dual-BCC phases. We also confirmed the presence of a dendritic structure corresponding to the XRD phase analysis. A chemical composition analysis showed the slight alloying elements’ segregation regarding their melting points. The increase in the microhardness was measured for Mo-containing samples, whereas the inverse phenomenon was observed for Hf-containing HEAs, where the microhardness decreased. However, the microhardness was higher in both cases than in human bone and conventional biomaterials. Previous studies have also shown the high corrosion resistance of both series of alloys. All the studied HEAs exhibited high break-down potential (E_BD_) in comparison to conventional biomaterials [27,28]. Due to that, the current manuscript presents a detailed comparative analysis of experimental results derived from studies on an alloy with variable Zr composition, placing these findings alongside previously published data on alloys with variable composition of Mo and Hf. It summarises the extensive research conducted to enhance the understanding of bio-compatible high-entropy alloys, focusing specifically on their structural and material properties. This research is part of a broader effort to advance the scientific knowledge base surrounding bio-HEA characteristics and applications in various fields.

## 2. Materials and Methods

The studied alloys’ compositions and their abbreviations are presented in Table 1. For all the studied alloys with various chemical composition, the thermodynamical parameters, such as atomic size mismatch (δ), mixing enthalpy (ΔH_mix_), mixing entropy (ΔS_mix_), electronegativity differences (Δχ), valence electron concentration (VEC), and Ω parameters, have been calculated (Table 1). The parameters have been discussed in our previous work [37]. All of the above-mentioned thermodynamical parameters are used for phase formation predictions for high-entropy materials. 

**Figure 1:**
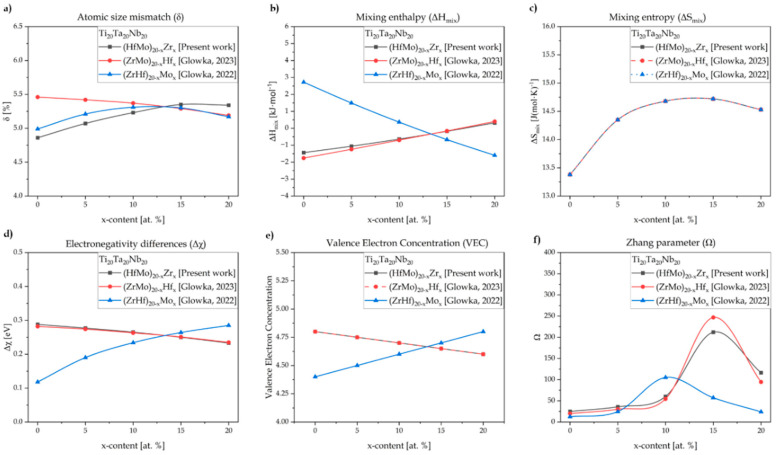
A comparison of the thermodynamical parameters: (**a**) atomic size mismatch (δ), (**b**) mixing enthalpy (ΔH_mix_), (**c**) mixing entropy (ΔS_mix_), (**d**) electronegativity differences (Δχ), (**e**) valence electron concentration (VEC), and (**f**) Zhang parameter (Ω) with previous literature-reported Hf- and Mo-containing HEAs [27,28].

Based on the obtained results, the increase in atomic size mismatch (δ) and mixing enthalpy (ΔH_mix_) with the increase in Zr content is observed. Moreover, the increase in the atomic size mismatch by up to 5% suggests the higher probability of forming a multi-phase system. The mixing entropy (ΔS_mix_) increased from 13.38 J·(mol·K)^−1^ for the Zr_0 sample up to 14.72 J·(mol·K)^−1^ for Zr_15 and further decreased down to 14.35 J·(mol·K)^−1^ for the Zr_20 high-entropy alloy. However, it should be underlined that all the studied materials could be classified as high-entropy materials based on the second configurational entropy-based definition [38]. The electronegativity differences and valence electron concentration revealed a decrease in the values of both parameters with increased Zr content. Additionally, the *BCC* phases were predicted for all the studied alloys based on the VEC parameter. The Zhang parameter predicted the presence of a multi-phase structure. A high correlation between the calculated thermodynamic parameters and the structure of the obtained alloys was observed. For the obtained Zr-containing samples, multi-phase *BCC* structures were revealed during an X-ray diffraction (XRD) phase analysis and observed on the recorded scanning electron microscopy (SEM) microstructure images, discussed further in the text. A comparison of the thermodynamic calculation of the above-presented parameters for Hf- and Mo-containing high-entropy alloys was also performed, and the graph is presented in Figure 1 [27,28]. 

The presented results show that for the atomic size mismatch (Figure 1a), the initial increase and further decrease were observed for the Zr- and Hf-containing samples. On the other hand, the Mo-containing HEAs exhibited an increase in the δ parameter. For mixing enthalpies (Figure 1b), the increase in this parameter was revealed for the Zr- and Hf-containing HEAs. An inverse phenomenon was observed for the Mo-containing materials. The similarity of mixing entropy (Figure 1c) was presented for all the studied and previously reported high-entropy materials. The performed thermodynamical calculations of electronegativity differences (Figure 1d) and valence electron concentration (Figure 1e) confirmed a decrease in both parameters for the Zr- and Hf-containing samples but an increase in the Mo-containing HEAs. For the Zhang parameter (Figure 1f), all the studied and literature-reported HEAs revealed an initial increase, with a further decrease in this parameter [27,28]. It should be underlined that the experimental investigations and obtained results directly confirm the thermodynamic calculations of the phase predictions in the presented work and for the Hf- and Mo-containing high-entropy materials.

All the studied Zr-containing HEAs were produced from elemental powders obtained by the air plasma spray (APS) technique delivered by Kamb Import–Export (Warsaw, Poland)—Nb, Ta, Ti, and Mo—and Atlantic Equipment Engineers (Upper Saddle River, NJ, USA)—Zr. A bulk rod of pure Hf with a diameter of d = 15 mm was used for mechanical grinding to obtain the powder form. The technological parameters of the powders mentioned above are presented in Table 2. 

**Figure 2:**
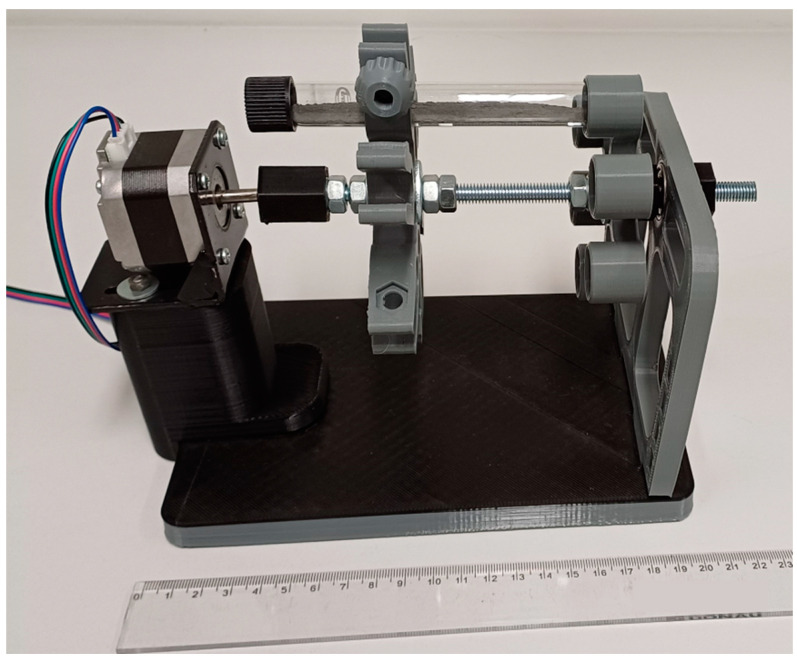
Three-dimensional-printed author-designed prototype, revolver-like machine for blending of elemental powders.

A Radwag AS 60/220/C/2 (Radom, Poland) laboratory balance was used to weigh each chemical element’s appropriate mass to obtain 5 g final samples. The elemental powders were blended for 72 h using the authors’ own designed prototype, a revolver-like 3D-printed machine equipped with an electric motor, to improve the homogeneity of the powders (Figure 2). 

Green compacts 10 mm in diameter were obtained for further melting by compression under a pressure of 8 tons. The bulk form of all the Zr-containing HEAs was obtained by the arc melting (AM) process in an argon atmosphere with a chamber pressure of 1.2 bar. Before the melting of the green compacts, a Ti-getter pellet was melted to capture residual gases in the chamber. Homogeneity of the melting buttons was ensured by re-melting 5 times by the sequence of preliminary melting for 120 s and 60 s mixing 4 times in the liquid state. All the Zr-containing HEAs were investigated in an as-cast state. 

Silica carbide (SiC) grinding papers (grit 360 to 2400), short-nap synthetic clothes, monocrystalline diamond suspensions from Buehler (Lake Bluff, IL, USA) with 6 μm to 1 μm particle sizes, a porous, neoprene MD-Chem polishing cloth from Stures (Ballerup, Denmark), and a silica dioxide (SiO_2_) colloidal suspension OP-S (Struers, Ballerup, Denmark) with 0.04 μm particle size were applied to prepare metallographic specimens from the as-cast alloys. The metallographic specimens were prepared using a Metkon Forcimat 1V grinding–polishing machine equipped with an automatic header Metkon Forcipol (Bursa, Turkey). 

Experimental X-ray diffraction (XRD) patterns were recorded in an angular range of 2θ = 20–130° with a 0.026° step in Bragg–Brentano scan geometry (θ–θ) at room temperature using a Panalytical Empyrean diffractometer (Malvern Instruments, Malvern, UK) equipped with a Cu anode (λ = 1.54056 Å wavelength). The operating electric current and voltage were 30 mA and 40 kV, respectively. Additionally, the XRD equipment included a solid-state hybrid, ultra-fast PIXcell^3D^ X-ray detector (Malvern Instruments, Malvern, UK). The Powley refinement method was applied to refine the lattice parameters using FullProf software (https://www.ill.eu/sites/fullprof/) [39].

Scanning electron microscopy (SEM) microstructure observations and X-ray spectroscopy energy-dispersive (SEM-EDS) chemical composition analysis were performed using a JEOL JSM-6480 (JEOL Ltd., Tokyo, Japan) scanning electron microscope equipped with an IXRF detector (IXRF, Austin, TX, USA). An accelerating voltage of 20 kV was applied to record microstructure microphotographs. ImageJ free-of-charge computer software (version: 1.51j8) was used to calculate the average percentage phase contribution from the recorded microstructure images. 

An electrochemical measurement was performed to determine the corrosion resistance behaviour of the studied Zr-containing HEAs. The sample preparation process included SiC sandpaper grinding (grit 800 to 2500) and polishing using a colloidal OP-S (Struers, Ballerup, Denmark) silica dioxide (SiO_2_) suspension and an MD-Chem cloth. Ringer’s solution (8.6 g/L NaCl, 0.3 g/L KCl, 0.48 g/L CaCl × 6H_2_O) simulated the body fluid environment. Before the measurements, argon with 99.999% purity was applied to deaerated Ringer’s solution. The measurements were carried out at 37(1) °C using Metrohm/Eco Chemie Autloab PGSTAT30 Potentiostat/Galvanostat Electrochemical System (Herisau, Switzerland). The research apparatus included a three-electrode electrochemical cell mounted in a Luggin’s capillary: (1) the working electrode (WE), which studied Zr-containing HEAs, (2) the platinum (Pt) counter electrode (CE), and (3) the saturated calomel electrode (SCE) as the reference electrode (RE). For the 10 min study, the HEAs were depassived at −1.2 V vs. SCE and further measured using open-circuit potential (E_OC_)—collected for 2 h, potentiodynamic polarisation (v = 2 mV s^−1^ sweep rate), and using electrochemical impedance spectroscopy (EIS) techniques. The EIS measurements were performed at E_OC_ with 10 frequencies per decade scanned using a sine-wave amplitude of 10 mV and a frequency range f = 50 kHz–1 mHz.

The mechanical properties of the obtained alloys were focused on the microhardness measurements, which were carried out using a MicroVickers tester 401MVD (Wilson Instruments, Norwood, MA, USA). A pyramid-shaped ~136° Vickers tip was applied for the microhardness measurements. During the investigation, 1 kgf (HV1) and a dwell time of 10 s were adjusted. For each sample, 20 indent areas were chosen, and the average microhardness was determined. 

## 3. Results and Discussion

### 3.1. XRD Phase Analysis of Studied High-Entropy Alloys

**Figure 3:**
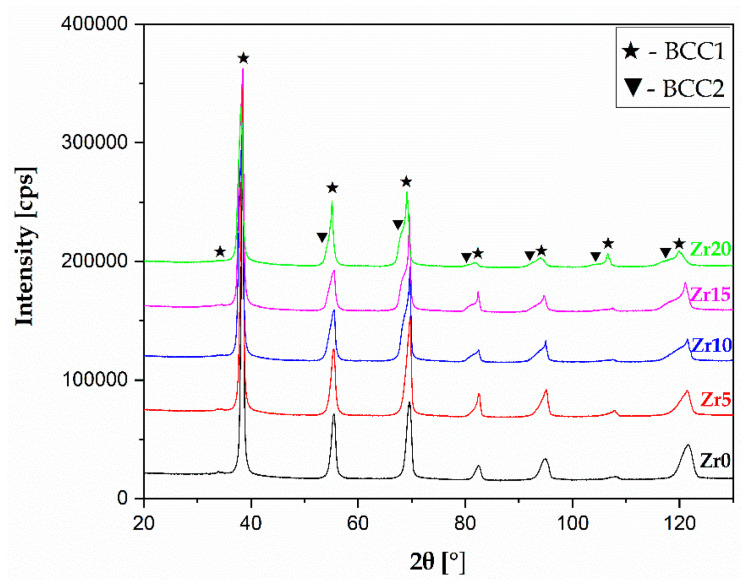
Diffraction patterns of the studied high-entropy alloys collected using the XRD technique.

The experimental XRD patterns with the phase marks for all the studied Zr-containing high-entropy alloys are presented in Figure 3. 

**Figure 4:**
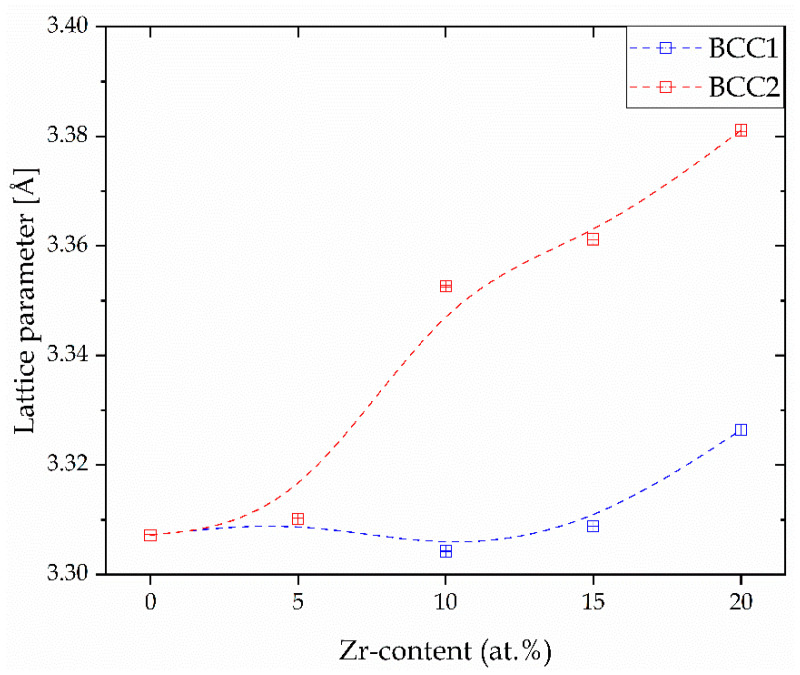
The variation in the unit cell parameters for *BCC1* and *BCC2* phases with the Zr content change for all HEAs after the Powley refinement.

An X-ray diffraction phase analysis confirmed the presence of diffraction peaks corresponding to two *BCC* phases (dual-*BCC*), denoted as *BCC1* and *BCC2*, respectively. The direct agreement between the XRD phase analysis and the thermodynamical calculations presented in Table 1 should be underlined. The obtained XRD measurements closely correlated to the predictions made based on atomic size mismatch (δ), VEC, and Zhang (Ω) parameters calculations. The calculations of both parameters predicted the formation of a multi-phase structure (Ω) with a *BCC*-type structure (VEC). Additionally, a similar phenomenon was observed for our previous literature-reported XRD phase analysis of Hf- and Mo-containing HEAs [27,28]. The dual-*BCC* phase is often visible in the literature for high-entropy alloys with similar chemical composition [33]. The XRD phase analysis also showed the differences in *BCC1*- and *BCC2*-phase lattice parameters, which were refined by Powley’s refinement and are presented in Table 3. Our previous works also showed slight differences in the lattice parameters of similar phases [27,28]. The obtained results are also graphically presented below (Figure 4). 

**Figure 5:**
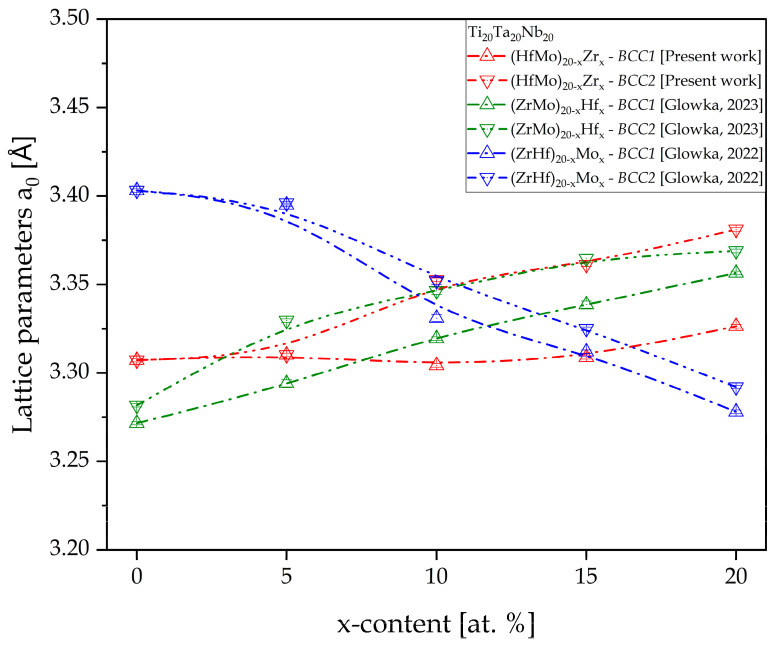
The comparison of the lattice parameters refined using Powley refinement with lattice parameters of Hf- and Mo-containing high-entropy alloys [27,28].

The obtained result reveals that the lattice parameters for both the *BCC1* and *BCC2* phases increased with the increase in the Zr content. The lattice parameters of both *BCC* phases were similar for the Zr_0 sample. It was also revealed that the lattice parameters of the *BCC2* phase were higher than for the *BCC1* phase, indicated by 2θ shift to the lower values. The differences in lattice parameters of both phases underlines a high agreement with the literature-reported data for bio-HEAs with similar chemical composition and our previous studies of HEAs with different Hf and Mo content [27,28,33]. Due to the largest atomic radii of the Zr element (r_iZr_ = 1.603 Å [27]), the increase in the lattice parameters with an increase in the Zr content was expected and experimentally confirmed. The highest atomic radii of the Zr contributed to the high level of the lattice distortion effect, which is one of the core effects of HEAs [40]. Moreover, the increase in the lattice parameters was also described in the literature for the ternary Ti-35Nb-XZr alloys, where the a_0_ parameter increases with the increase in the Zr content [41]. A similar phenomenon was also confirmed in our previous studies of Hf-containing HEAs due to the second large atomic radii of the Hf [28]. Additionally, the above-presented refined lattice parameters were compared with our previous data for high-entropy alloys with different Hf and Mo content as reported in [27,28] and presented in Figure 5. It is worth noting that the presented curves cross each other close to x = 0.13, which would be a composition with the equiatomic concentration of the Zr, Hf, and Mo elements.

The obtained result reveals that only for Mo-containing bio-HEAs, the decrease in the lattice parameters for both the *BCC* phases was confirmed [27]. As it was mentioned above, for the Zr- and Hf-containing bio-HEAs, the inverse phenomenon was observed [28]. For the *BCC1* phase, it was confirmed that the lowest lattice parameters were confirmed in the Hf_0 sample a_0_ = 3.2716(1) Å, and the largest lattice parameter of this phase was confirmed in the Mo_0 sample (a_0_ = 3.4031(1) Å) [27,28]. On the other hand, for the *BCC2* phase, the smallest a_0_ was revealed in the Hf_0 sample (a_0_ = 3.2817(1) Å), but the highest was in the Mo_5 sample a_0_ = 3.3960(1) Å [27,28]. The above-presented results underline the highest impact of the different Hf and Mo content on the lattice parameters of the studied materials. 

### 3.2. SEM Microstructure and EDS Chemical Composition Analysis of Studied High-Entropy Alloys

The morphology of the elemental powders recorded on the secondary electron contrast images (SEI) was previously presented in the bio-HEAs with different Mo content [27]. 

**Figure 6:**
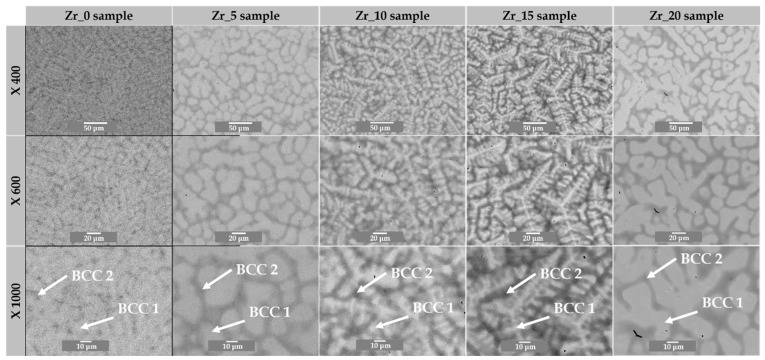
SEM microstructure images recorded using backscattered electron contrast (BSE) of studied materials with denoted *BCC1* and *BCC2* regions.

According to the thermodynamical parameter calculations, the Zhang parameter (Ω) predicted the formation of a multi-phase structure for all the investigated materials (see Table 1). The recorded microstructure images using backscattered electron contrast (BSE) confirmed the calculations of the parameters, and the presence of two phases was revealed. The phases denoted as *BCC1* and *BCC2* corresponded to the dendritic and interdendritic regions, respectively (Figure 6). It should be underlined that the microstructure presented in the current work is similar to the microstructure of high-entropy alloys with different Hf and Mo content, described in our published work [27,28]. Moreover, the recorded micrograph stays in high agreement with the XRD phase analysis, where the presence of diffraction of peaks corresponding to the dual-*BCC* phases was confirmed (earlier in the text and Figure 3). 

According to the recorded SEM images, the smallest chemical contrast (corresponding to the atomic number (Z)) between the *BCC1* and *BCC2* phases was observed in the Zr_0 sample. For the Zr_5 and Zr_20 samples, the dendritic phase’s elongation was revealed compared to the Zr_10 and Zr_15 samples, where a characteristic dendritic structure was confirmed. For all the studied HEAs, the separation of the *BCC2* phase corresponding to the interdendritic region from the dendritic region was also observed and corresponds to the grain boundary wetting phenomena described by Cahn and Straumal et al. [42,43,44,45]. Our previous work also observed grain boundary wetting for different Mo-containing high-entropy alloys [27]. 

Based on the recorded micrographs, each phase average percentage contribution was calculated using the image processing computer software and is presented below (Table 4). The average percentage contribution of the phases was determined based on the four microstructure micrographs for each sample (the summarised surface area was 0.14 mm^2^).

**Figure 7:**
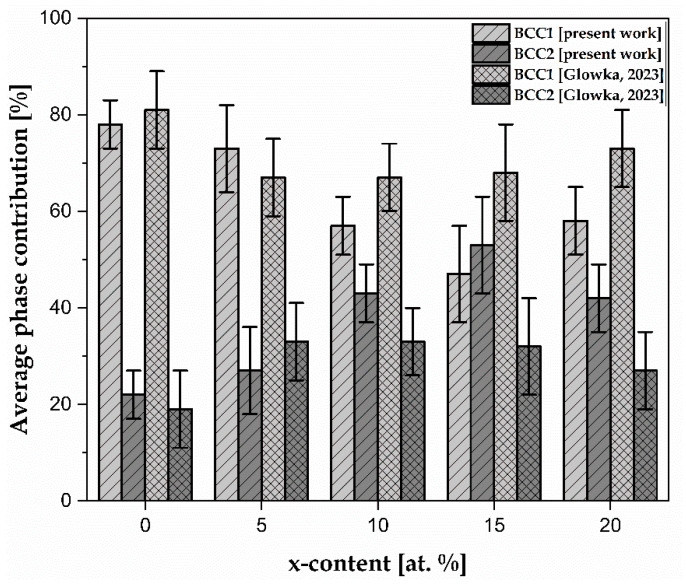
The average phase contribution of the *BCC1* and *BCC2* with the literature-reported Hf-containing HEAs [28].

According to the results above, it can be confirmed that the average contribution of the *BCC2* phase increased up to 15% of the Zr concentration from 23(5) to 56(10) %. However, a further decrease in this phase was revealed. The average phase contribution was also compared with the average phase contribution calculated for the literature-reported Hf-containing HEAs (Figure 7) [28]. 

The results reveal that the average phase contribution of *BCC1* for Zr_0, Zr_10, Zr_15, and Zr_20 was smaller than that of the Hf-containing samples with similar concentrations [28]. On the other hand, only for the Zr_5 sample was the increase in the contribution of the *BCC1* phase measured compared with the *BCC1* phase for the Hf_5 sample [28]. Analogously, a higher contribution of the *BCC2* phase was observed for the Zr_0, Zr_10, Zr_15, and Zr_20 samples, but it was smaller for Zr_5 than for the Hf-containing materials [28]. 

**Figure 8:**
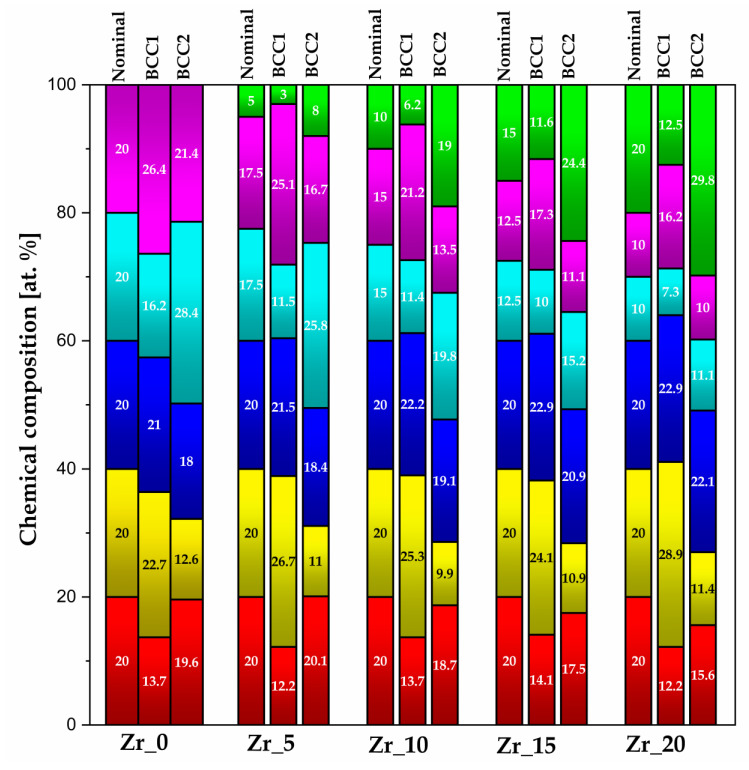
Graphical presentation of the SEM-EDS chemical composition analysis (colour bars: red—Ti, yellow—Ta, blue—Nb, light blue—Hf, purple—Mo, and green—Zr). Moreover, colour bars correspond to the elemental distribution maps (Figure 9).

**Figure 9:**
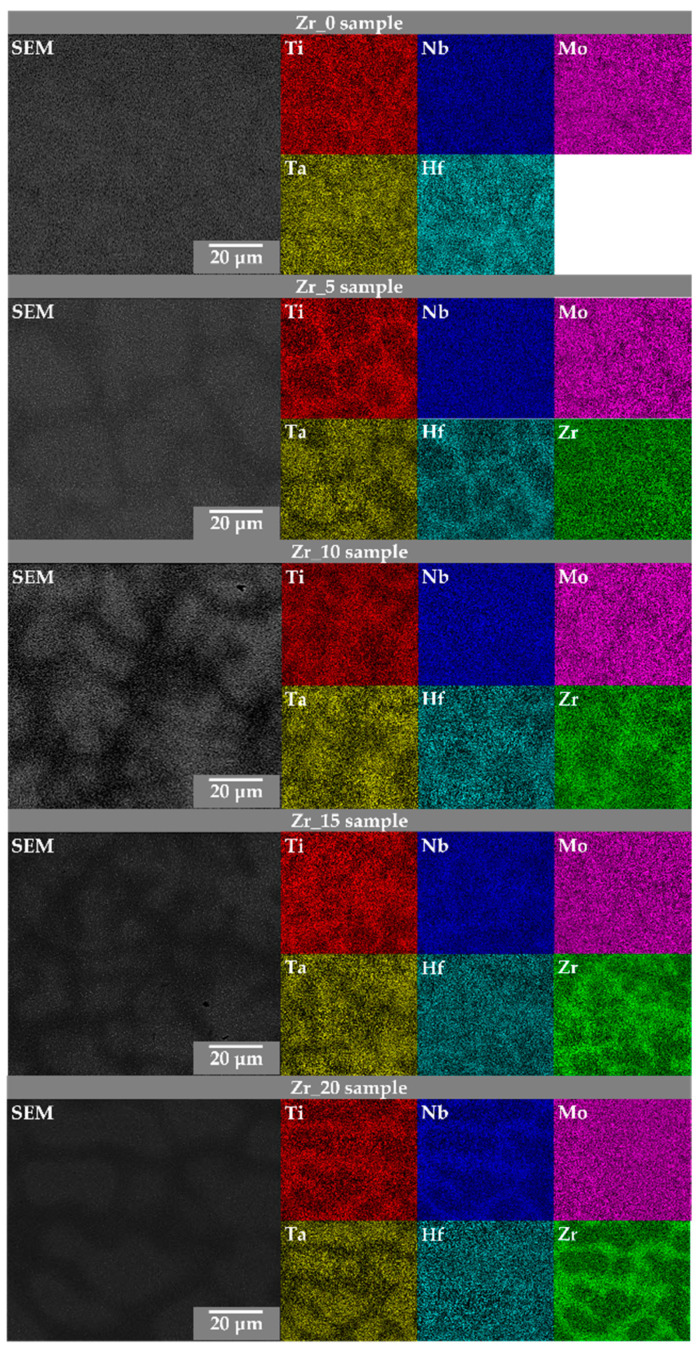
Elemental distribution maps (EDMs) showing relative chemical composition recorded using the SEM-EDS technique.

The average chemical composition of the *BCC1* and *BCC2* phases was calculated based on 40 areas for each phase and is presented in Figure 8. The colours of the bars correspond to the elemental distribution maps (EDMs) presented in Figure 9.

The as-cast state of the studied high-entropy alloys contributed to the segregation of the alloying, similar to our previous results of different Hf and Mo content [27,28]. For all the studied HEAs samples, the elemental segregation according to the melting point of the alloying elements was observed. Due to that, the *BCC1* phase was mainly Ta-, Nb-, and Mo-enriched due to their higher melting temperature in comparison to Ti, Hf, and Zr, mainly located in the *BCC2* regions. The colours of the bars correspond to the elemental distribution maps (EDMs) presented in Figure 8. 

Due to the alloying elements’ segregations in the microstructure, confirmed in Figure 8, the SEM-EDS intensity elemental distribution maps were also recorded (Figure 9). 

According to the presented results, it can be concluded that for the samples with the lowest Zr, Hf, and Mo concentrations (0 at.%), the *BCC1* phases were mainly Ta-, Nb-, and Mo-enriched (Zr_0 and Hf_0 samples). However, for the Mo_0 sample, this phase was Ti-, Nb-, and Zr-enriched. The comparison of the chemical composition of the *BCC2* phases revealed higher concentrations of Ti and Hf for the Zr_0 sample, Zr and Nb for the Hf_0 sample, and Ti, Nb, and Zr for the Mo_0 sample [27,28]. 

The samples with 5 at.% of Zr, Hf, and Mo content revealed a higher Ta, Nb, and Mo concentration in the *BCC1* phases for the Zr_5 and Hf_5 samples. Moreover, the Mo_5 sample confirmed Ti, Ta, Nb, Hf, and Zr enrichment. For the *BCC2* phase, the highest Ti, Hf, and Zr concentration was confirmed for all the studied Zr_5, Hf_5, and Mo_5 samples [27,28]. 

The samples with 10 at.% of Zr, Hf, and Mo concentration confirmed the main enrichment of the *BCC1* phases in Ta, Nb, and Mo (Zr_10 and Hf_10 samples). Additionally, for the Mo_10 sample, a higher Ti content was also present. For *BCC2* phases, all the studied materials confirmed Ti, Hf, and Zr enrichment. It should be underlined that such phenomena were also confirmed for the chemical composition of the *BCC2* phases for samples with 5% of Zr, Hf, and Mo [27,28].

For the Zr_15, Hf_15, and Mo_15 samples, the chemical composition of the *BCC1* phases confirmed similar results compared to the above-described chemical composition analysis of the Zr_10, Hf_10, and Mo_10 samples. For the *BCC1* phases, higher Ta, Nb, and Mo content was observed. However, the enrichment in Hf and Ti was also revealed in the Hf_15 and Mo_15 samples, respectively. The chemical composition analysis of the *BCC2* phases confirmed the similarity to the EDS chemical composition of the samples with 5% and 10% of Zr, Hf, and Mo. The investigated *BCC2* confirmed Ti, Hf, and Zr enrichment [27,28].

For the samples with the highest Zr, Hf, and Mo content (20 at.%), the SEM-EDS analysis confirmed the enrichment in Ta, Nb, and Mo in the Zr_20 and Mo_20 samples in the *BCC1* phases. Additionally, the *BCC1* phase for the Hf_20 sample was Ta- and Hf-enriched. For *BCC2*, Ti, Hf, and Zr enrichment phenomena were confirmed for the Zr_20 and Mo_20 samples. Furthermore, the *BCC2* phase of the Hf_20 sample also confirmed Nb enrichment [27,28]. 

### 3.3. Microhardness of Investigated HEAs

Microhardness tests measured the influence of the Zr/(HfMo) ratio to determine the selected mechanical properties of the obtained high-entropy materials. As it was mentioned in the Materials and Methods section of the manuscript, for each Zr-containing sample, 20 indent areas were chosen. Nevertheless, the micrometric sizes of the pyramidal Vicker’s tip contributed to the calculations of the average microhardness for the *BCC1* and *BCC2* phases (Table 5). 

**Figure 10:**
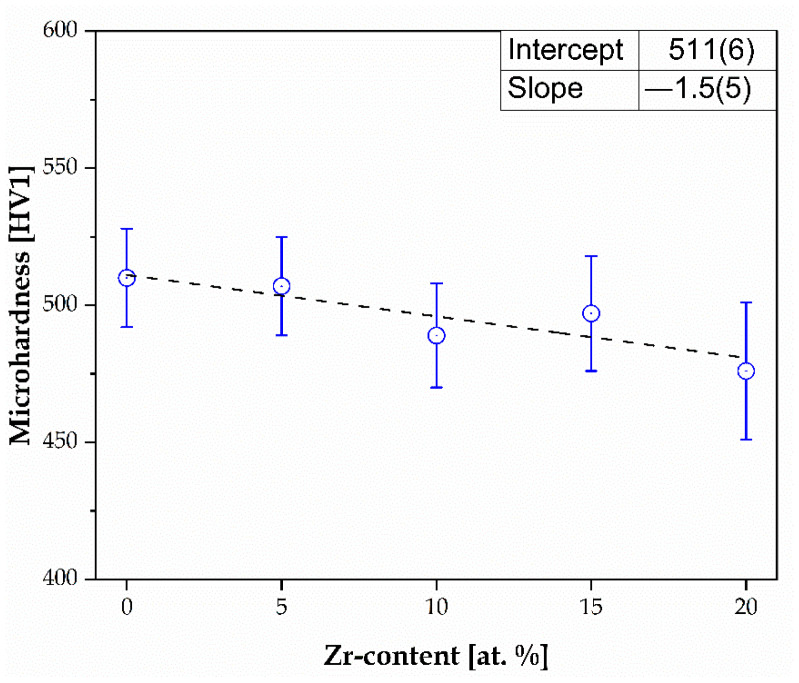
Microhardness of studied Zr-containing bio-HEAs with a linear fit.

A graphical presentation of the dependence of the Zr/(HfMo) ratio on the microhardness is presented in Figure 10.

The decrease in the microhardness could be provoked by the microstructure evolution, especially in the case of the Hall–Petch relation between the hardness and average grain size. Based on the SEM microstructure analysis (Figure 6) and phase contribution (Figure 7 and Table 4), it was calculated that the phase contribution of the dendrites (*BCC1* phase) decreased, but the contribution of the interdendritic phase increased (*BCC2* phase). This phase contribution increase could contribute to the phase growth, which increases the microhardness. However, a detailed analysis of this phenomenon needs to be undertaken. 

Moreover, the microhardness of the studied Zr-containing HEAs was compared with our Hf- and Mo-containing bio-HEAs with similar chemical composition, conventional biomaterials, and human bone (Table 6). 

According to the presented results, the microhardness of all the studied Zr-containing HEAs was comparable with the literature reporting Hf- and Mo-containing HEAs [27,28]. Nevertheless, the microhardness was higher in comparison to Ti-based biomaterials and human bone. However, only the microhardness of the Zr_20 sample was slightly higher compared to the additive-manufactured surgical stainless steel. On the other hand, the microstructure of the studied HEAs was lower than NiTi after the deformation process. 

**Figure 11:**
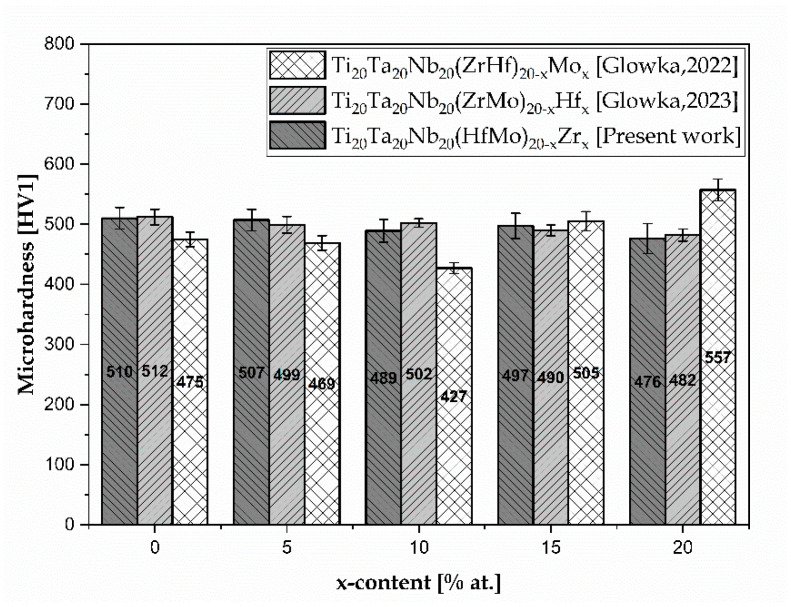
Comparison of microhardness with literature-reported biomedical high-entropy alloys with different Hf and Mo concentrations [27,28].

Additionally, the influence of Zr addition on microhardness was compared with the influence of Hf and Mo, as characterised in previous publications, and is presented in Figure 11 [27,28].

According to the above-presented data, it can be concluded that the microhardness of the studied Zr-containing HEAs was comparable with the microhardness of high-entropy alloys with different Hf content [28]. On the other hand, for the Zr_0, Zr_5 and Zr_10 samples, the microhardness was higher compared to the Mo-containing HEAs [27]. Additionally, the Zr_15 sample exhibited a decrease in microhardness compared to the Mo_15 sample [27]. For the Zr_20 sample, the microhardness was the smallest in comparison to the Hf- and Mo-containing materials with similar chemical composition [27,28]. 

### 3.4. Corrosion Resistance of Investigated HEAs

**Figure 12:**
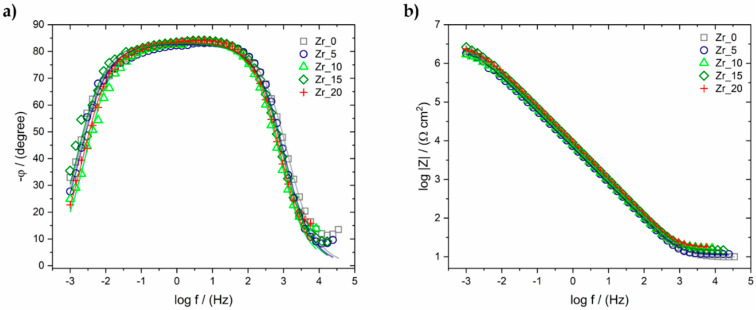
(**a**) Bode diagram registered at E_OC_ and (**b**) log |Z| = f(log f) curves exposed in Ringer’s solution at 37 °C.

Electrochemical measurements were performed in Ringer’s solution to characterise the corrosion resistance of the investigated alloys. The open-circuit potential measurements were carried out for 2 h for all the Zr-containing samples. During this period, the value of the E_OC_ was stabilised, and the results are summarised in Table 8. The results obtained for all the investigated samples of high-entropy alloy differ slightly and fluctuate between the values of −271 and −314 mV. There is no visible tendency for the E_OC_ values to shift towards positive or negative potentials with increasing Zr content. This result might suggest that the Zr content does not significantly influence corrosion resistance. The results of the AC impedance measurements are presented in the form of a Bode diagram in Figure 12. 

Based on the results of the EIS, an oxide layer could be assumed to be present on the surface of the investigated alloys. Such phenomenon may be evidenced by a one-time constant, a broad plateau in the range of medium frequencies (0.01–100 Hz) visible on the graph (Figure 12a), which indicates passive protection of the investigated material. Figure 12b displays the log |Z| in a function of the logarithm of the measuring frequency. The slope of log |Z| indicates the captive character of the passive film. The value of log |Z|_f=0.01 Hz_ at low frequencies corresponds to the material’s resistance to pitting corrosion. A slight increase in the value of log |Z|_f=0.01 Hz_ with an increase in the Zr content in the high-entropy alloy was observed, which may indicate an improvement in the resistance of the studied materials to pitting corrosion.

**Figure 13:**
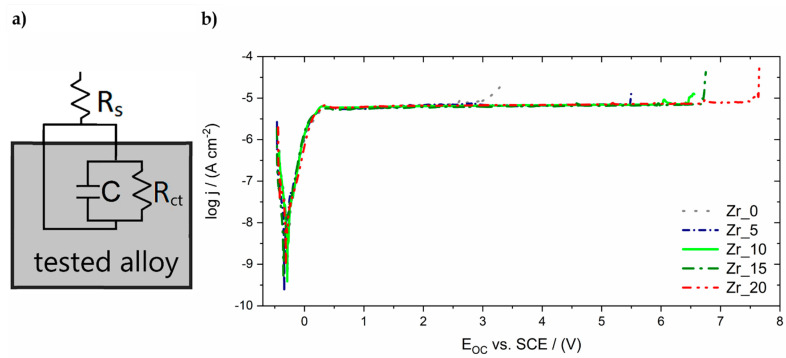
(**a**) Randle’s equivalent circuit model and (**b**) anodic polarisation curves exposed in Ringer’s solution at 37 °C.

The experimental EIS data were analysed based on the concept of equivalent electrical circuits (EEC) concerning the physical meaning of the used circuit elements. The EQUIVCRT program was used for calculation. The Randle’s cell (Figure 13a), which represents the physical model of the oxide layer–Ringer’s solution system, was utilised for the obtained data. R_s_ represents the resistance of the solution; R_ct_ is the resistance of charge transfer through the oxide layer–Ringer’s solution interface; and C is the electrical double-layer capacitance parameter, corresponding to an ideal capacitor. The constant phase element (CPE) was used instead of a capacitor for the approximation procedure. The error of the particular parameter determination was consistently below 3%. The results are presented on Bode plots in the form of continuous lines and summarised in Table 7. 

The values of the R_ct_, which characterises the resistance of the oxide layer presented on the surface of the alloys, varied from 1.74 × 10^6^ up to 3.19 × 10^6^ Ω·cm^2^ depending on the Zr content. The obtained results indicate very good corrosion resistance of the studied Zr-containing bio-HEAs. This corresponds to the calculated low value of the double-layer capacitance.

The registered potentiodynamic curves exhibited a similar shape to a broad plateau, which indicates the protective properties of the oxide layer and can be related to the transpassivation process (Figure 13b).

The passive range varies depending on the investigated sample. The end of the passive ranges was observed with a significant increase in the current density. This phenomenon is correlated with the break-down of the protection oxide layer and the dissolution of the surface. The summarised results confirm that the break-down potential value (E_BD_) increased with the increase in the Zr amount (Table 8). The sample with the highest Zr content exhibited the best corrosion resistance due to the anodic dissolution of the passive layer starting above 7.60 V. On the other hand, the break-down of the oxide layer started around 3.00 V for the sample without a Zr concentration. 

Additionally, the obtained results were compared with the previous literature-reported biomedical high-entropy alloys with different Hf and Mo content and commercial biomaterials (Table 8). 

Based on the obtained results, it should be underlined that for the Zr_5, Zr_10, Zr_15, and Zr_20 samples, the break-down potential was the highest in comparison to all the presented data for the high-entropy alloys and commercial biomaterials. The E_BD_ = ~3.00 V vs. SCE was lower than the Hf- and Mo-containing bio-HEAs and biomedical Ti-15Mo alloy only for the Zr_0 sample. However, the break-down potential of this sample was still higher compared to the Titanium Grade 7, Ti-6Al-4V, cp-Ti Grade 2, Ti-6Al-7Nb, Ti-13Nb-13Zr, 316L SS, pure Ti, NiTi, and Ti-45Nb biomaterials. Electrochemical measurements underlined the high impact of the Zr on the corrosion resistance in the presented high-entropy alloys. Moreover, the literature data also underlines that the oxide layers of the alloying elements TiO_2_, Ta_2_O_5_, Nb_2_O_5_, ZrO_2_, HfO_2_, and MoO_2_ also ensured the high corrosion resistance of the studied materials [32,62]. 

**Figure 14:**
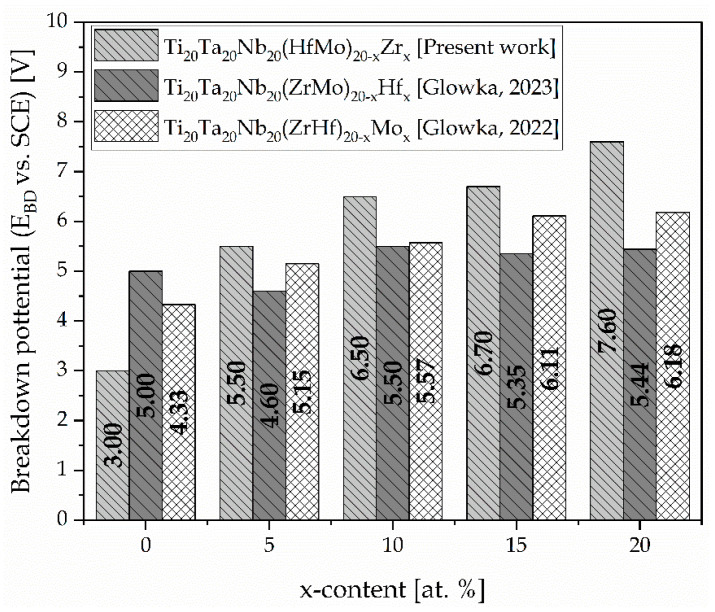
The comparison of the break-down potential (E_BD_) with previously reported Hf- and Mo-containing HEAs [27,28].

The obtained break-down potential (E_BD_) was compared with those previously reported for the Hf- and Mo-containing HEAs and is graphically presented below (Figure 14) [27,28]. 

According to the above-presented results, the Zr content contributed to a significant increase in the break-down potential. It was confirmed that the E_BD_ of the Zr_5, Zr_10, Zr_15, and Zr_20 bio-HEAs was the highest compared to the previously reported break-down potential for the Hf- and Mo-containing bio-HEAs [28,29]. However, the sample without Zr content (Zr_0) presented the lowest break-down potential compared to all the samples. 

## 4. Conclusions

In the presented study, the influence of Zr/(HfMo) was investigated for the six-elemental Ti_20_Ta_20_Nb_20_(HfMo)_20−x_Zr_x_ (where x = 0, 5, 10, 15, and 20 at.%) high-entropy alloys in an as-cast state, produced from powders by the arc-melting technique. Additionally, the obtained results were compared with those previously presented in the literature for Hf- and Mo-containing HEAs. 

The experimental process directly confirmed the correctness of the thermodynamic calculations of the phase formation parameters. The multi-phase microstructure composed of *BCC* phases expected based on the calculations was observed on the collected XRD patterns. Similar dual-*BCC* phases were observed for the previously reported Hf- and Mo-containing HEAs. The published results also show direct agreement between the thermodynamical calculations and phase analysis results. The slight differences in the lattice parameters were determined for the Zr-, Hf-, and Mo-containing alloys. Moreover, the increase in Zr content increased the lattice parameter due to the highest atomic radii of the Zr element. The exact correlation was observed for the literature-described ternary Ti-Nb-Zr alloys. Additionally, a similar phenomenon was observed for our previously characterised Hf-containing bio-HEAs. Only for the Mo-containing HEAs was the decrease in the lattice parameters determined. The highest impact on the lattice parameters of *BCC1* and *BCC2* was observed for the Hf- and Mo-containing HEAs.

The BSE micrographs show that the microstructure of all the studied alloys is composed of dendritic and interdendritic regions. The observed microstructure corresponds to the dual-*BCC* phases identified during the XRD phase analysis and predicted by the atomic size mismatch (δ), VEC (*BCC*), and Ω (multi-phase) parameters. The phase contribution corresponding to the literature-reported wetting phenomena reveals the higher contribution of dendritic (*BCC1*) phases compared to the interdendritic region (*BCC2*). However, the opposite phenomenon was observed for 15% of the Zr addition. Compared to the literature-described Hf-containing HEAs, a smaller contribution of the *BCC1* phase was observed for the samples with 0, 10, 15, and 20 at.% of Zr content.

The segregation of the alloying elements based on the melting temperatures was also observed using the SEM-EDS method and presented by elemental distribution maps (EDMs). The obtained SEM-EDS exhibits high agreement with previous results for Hf- and Mo-containing HEAs. The chemical composition analysis of the *BCC1* phases confirms the enrichment mainly in Ta, Nb, and Mo for all the studied Zr-, Hf- and Mo-containing samples. However, the higher content of Ti and Hf was also observed for single samples. On the other hand, the chemical compositions of the *BCC2* phases revealed prominent enrichment in Ti, Hf, and Zr, as well as in the Nb. 

The microhardness of the studied Zr-containing HEAs decreased from 510(18) to 476(25) HV1 with increased Zr content. Additionally, the obtained microhardness was comparable with the previously reported microhardness of Hf-containing HEAs. Furthermore, the microhardness of the five-elemental Ti_20_Ta_20_Nb_20_Hf_20_Mo_20_ (Zr_0), Zr_5, and Zr_10 samples was higher than the Mo-containing HEAs. Approximately 15% of the Zr addition contributed to the decrease in the microhardness compared to the Mo-containing sample with the same concentration. It should be underlined that the lowest microhardness compared to the literature-reported Hf- and Mo-containing HEAs was measured for the sample with 20% Zr addition. Unfortunately, the microhardness is still high compared to Ti-based conventional biomaterials. 

The electrochemical characteristic of the studied materials in Ringer’s solution confirms the significant impact of the Zr addition on the break-down potential (E_BD_) of the oxide layer. The obtained results vary from 3.00 V vs. SCE to 7.60 V vs. SCE for the Zr_0 and Zr_20 samples, respectively. It should be underlined that the increase in the Zr content from 5 at.% up to 20 at.% contributed to the increase in the E_BD_. Furthermore, the obtained results for these samples were the highest compared to the previous literature-reported Hf- and Mo-containing bio-HEAs and conventional biomaterials. However, the break-down potential of the Zr_0 sample was lower than the Hf- and Mo-containing bio-HEAs and Ti15Mo biomaterial but still higher compared to the selected biomaterials, such as Ti6Al4V, pure Ti, and NiTi. 

## Figures and Tables

**Table 1:** Thermodynamical parameters calculated for the studied Zr-containing HEAs: δ—atomic size mismatch, ΔH_mix_—mixing enthalpy, ΔS_mix_—mixing entropy, Δχ—electronegativity differences, VEC—valence electron concentration, Ω—Zhang parameter, *BCC*—body-centred cubic.

Chemical Composition	Abbreviation	δ [%]	ΔH_mix_ [kJ∙mol^−1^]	ΔS_mix_ [J·(mol·K)^−1^]	Δχ [eV]	VEC	Ω
Ti_20_Ta_20_Nb_20_(HfMo)_20_	Zr_0	4.86	−1.44	13.38	0.288	4.80 (*BCC*)	24.87 (Multi-phase)
Ti_20_Ta_20_Nb_20_(HfMo)_17.5_Zr_5_	Zr_5	5.07	−1.06	14.35	0.277	4.75 (*BCC*)	35.84 (Multi-phase)
Ti_20_Ta_20_Nb_20_(HfMo)_15_Zr_10_	Zr_10	5.23	−0.64	14.68	0.265	4.70 (*BCC*)	60.06 (Multi-phase)
Ti_20_Ta_20_Nb_20_(HfMo)_12.5_Zr_15_	Zr_15	5.35	−0.18	14.72	0.250	4.65 (*BCC*)	211.97 (Multi-phase)
Ti_20_Ta_20_Nb_20_(HfMo)_10_Zr_20_	Zr_20	5.43	0.32	14.53	0.233	4.60 (*BCC*)	116.34 (Multi-phase)

**Table 2:** Technological parameters of elemental powders and bulk Hf.

Element	Deliverer	Purity [%]	Particle Size [μm]
Nb	Kamb Import–Export	99.9	70–180
Ta		99.9	<100
Ti		99.9	<90
Mo		99.5	<90
Zr	Atlantic Equipment Engineers	99.5	<250
Hf	Bulk rod	>99.9	98 (56) [28]

**Table 3:** Powley’s refined lattice parameters based on the collected XRD data.

Sample	Phase	Lattice Parameters a_0_ [Å]
Zr_0	*BCC1*	3.3072(1)
	*BCC2*	3.3072(1)
Zr_5	*BCC1*	3.3102(1)
	*BCC2*	3.3102(1)
Zr_10	*BCC1*	3.3088(1)
	*BCC2*	3.3527(1)
Zr_15	*BCC1*	3.3088(1)
	*BCC2*	3.3612(1)
Zr_20	*BCC1*	3.3264(1)
	*BCC2*	3.3811(1)

**Table 4:** Phase contribution of *BCC1* and *BCC2* phases.

Sample	Phase	Phase Contribution [%]
Zr_0	*BCC1*	78(5)
	*BCC2*	22(5)
Zr_5	*BCC1*	73(9)
	*BCC2*	27(9)
Zr_10	*BCC1*	57(6)
	*BCC2*	43(6)
Zr_15	*BCC1*	47(10)
	*BCC2*	53(10)
Zr_20	*BCC1*	58(7)
	*BCC2*	42(7)

**Table 5:** Average microhardness (HV1) of the studied biomedical high-entropy alloys.

Sample	Microhardness [HV1]
Zr_0	510(18)
Zr_5	507(18)
Zr_10	489(19)
Zr_15	497(21)
Zr_20	476(25)

**Table 6:** Comparison of microhardness of Zr-containing HEAs with literature-described Hf- and Mo-containing biomedical high-entropy materials, conventional biomaterials, and human bone.

Sample	Microhardness [HV1]	Reference
Zr_0	510(18)	Present work
Zr_5	507(18)	Present work
Zr_10	489(19)	Present work
Zr_15	497(21)	Present work
Zr_20	476(25)	Present work
Ti_20_Ta_20_Nb_20_(ZrMo)_20_	512	[28]
Ti_20_Ta_20_Nb_20_(ZrMo)_15_Hf_10_	502	
Ti_20_Ta_20_Nb_20_(ZrMo)_17.5_Hf_5_	499	
Ti_20_Ta_20_Nb_20_(ZrMo)_12.5_Hf_15_	490	
Ti_20_Ta_20_Nb_20_(ZrMo)_10_Hf_20_	482	
Ti_20_Ta_20_Nb_20_ (ZrHf)_10_Mo_20_	557	[27]
Ti_20_Ta_20_Nb_20_ (ZrHf)_12.5_Mo_15_	505	
Ti_20_Ta_20_Nb_20_(ZrHf)_20_	475	
Ti_20_Ta_20_Nb_20_(ZrHf)_17.5_Mo_5_	469	
Ti_20_Ta_20_Nb_20_ (ZrHf)_15_Mo_10_	427	
NiTi (hot-pressed and rolled)	693	[46]
316L SS (laser-cladded)	467.8	[47]
Ti-6Al-4V (selective laser-melted)	390	[48]
Ti-40Nb-10Ag (sintered at 975 °C)	360.4	[49]
Titanium Grade 4 (after SPD process)	330	[50]
cp-Ti (after high-pressure torsion)	305	[51]
316L SS (additive-manufactured)	300	[52]
Lamellar bone	88.8	[53]

**Table 7:** Summary of the parameters obtained using Randle’s equivalent circuit model.

Sample	R_s_ [Ω·cm^2^]	R_ct_ [Ω·cm^2^]	T [F·cm^−2^∙s^ϕ−1^]	ϕ	C [F·cm^−2^]
Zr_0	9.88	2.53 × 10^6^	0.99 × 10^−5^	0.918	4.34 × 10^−6^
Zr_5	11.10	2.14 × 10^6^	0.11 × 10^−4^	0.921	5.08 × 10^−6^
Zr_10	16.00	1.74 × 10^6^	0.11 × 10^−4^	0.927	5.57 × 10^−6^
Zr_15	14.61	3.19 × 10^6^	0.92 × 10^−5^	0.925	4.47 × 10^−6^
Zr_20	17.23	2.41 × 10^6^	0.91 × 10^−5^	0.931	4.75 × 10^−6^

**Table 8:** Registered open-circuit potential (E_OC_), the log|Z|_f→0.01Hz_, and break-down potential (E_BD_) for Zr-containing HEAs and the comparison of obtained E_BD_ with literature-reported Hf- and Mo-containing high-entropy alloys and conventional biomaterials.

Sample	E_OC_ vs. SCE [mV]	log|Z|_f→0.01Hz_ [Ω∙cm^2^]	E_BD_ vs. SCE [V]	Reference
Zr_0	−271	5.70	~3.00	Present work
Zr_5	−313	5.67	5.50	Present work
Zr_10	−278	5.73	6.50	Present work
Zr_15	−314	5.77	6.70	Present work
Zr_20	−294	5.84	7.60	Present work
Ti_20_Ta_20_Nb_20_(ZrHf)_10_Mo_20_	−333	6.28	~6.18	[27]
Ti_20_Ta_20_Nb_20_(ZrHf)_12.5_Mo_15_	−365	6.39	~6.11	
Ti_20_Ta_20_Nb_20_(ZrHf)_15_Mo_10_	−195	5.84	5.57	
Ti15Mo	―	―	5.50	[54]
Ti_20_Ta_20_Nb_20_(ZrMo)_15_Hf_10_	−199	5.50	~5.50	[28]
Ti_20_Ta_20_Nb_20_(ZrMo)_10_Hf_20_	−139	5.64	~5.45	
Ti_20_Ta_20_Nb_20_(ZrMo)_12.5_Hf_15_	−170	5.59	~5.35	
Ti_20_Ta_20_Nb_20_(ZrHf)_17.5_Mo_5_	−142	5.40	5.15	[27]
Ti-Nb-Zr-Ta	―	―	5.00	[55]
Ti_20_Ta_20_Nb_20_(ZrMo)_20_	−228	6.05	~5.00	[28]
Ti_20_Ta_20_Nb_20_(ZrMo)_17.5_Hf_5_	−270	5.54	~4.60	
Ti-15Mo	―	―	4.51	[56]
Ti_20_Ta_20_Nb_20_(ZrHf)_20_	−423	4.93	4.33	[27]
Titanium Grade 7	―	―	2.40	[57]
Ti-6Al-4V	―	―	1.53	[58]
Cp-Ti Grade 2	―	―	1.48	
Ti-6Al-7Nb	―	―	1.38	
Ti-13Nb-13Zr	―	―	1.25	
316L stainless steel	―	―	0.96	[59]
Pure Ti	―	―	0.50	[57]
Ti-15Nb	―	―	0.45	[60]
NiTi SMA	―	―	0.45	[61]
Ti-45Nb	―	―	0.28	[60]

## Data Availability

Data are contained within the article.
